# Quantifying Parameter Interdependence in Stochastic Discrete Models of Biochemical Systems

**DOI:** 10.3390/e25081168

**Published:** 2023-08-05

**Authors:** Samaneh Gholami, Silvana Ilie

**Affiliations:** Department of Mathematics, Toronto Metropolitan University, Toronto, ON M5B 2K3, Canada; silvana@torontomu.ca

**Keywords:** stochastic simulation algorithm, stochastic biochemical systems, sensitivity analysis, finite-difference methods, parameter subset selection, estimability analysis

## Abstract

Stochastic modeling of biochemical processes at the cellular level has been the subject of intense research in recent years. The Chemical Master Equation is a broadly utilized stochastic discrete model of such processes. Numerous important biochemical systems consist of many species subject to many reactions. As a result, their mathematical models depend on many parameters. In applications, some of the model parameters may be unknown, so their values need to be estimated from the experimental data. However, the problem of parameter value inference can be quite challenging, especially in the stochastic setting. To estimate accurately the values of a subset of parameters, the system should be sensitive with respect to variations in each of these parameters and they should not be correlated. In this paper, we propose a technique for detecting collinearity among models’ parameters and we apply this method for selecting subsets of parameters that can be estimated from the available data. The analysis relies on finite-difference sensitivity estimations and the singular value decomposition of the sensitivity matrix. We illustrated the advantages of the proposed method by successfully testing it on several models of biochemical systems of practical interest.

## 1. Introduction

Mathematical and computational modeling have become widespread in the study of complex dynamical systems, particularly in investigating cellular processes and biochemical networks [1]. Frequently, mathematical modeling of chemical reaction systems relies on deterministic differential equations and mass action kinetics. However, biochemical systems in the cell are intrinsically noisy [2,3], and thus stochastic models must be employed to account for the random fluctuations observed experimentally, especially when some species have low molecular counts [4,5]. One of the most popular stochastic discrete models of biochemically reacting systems is the Chemical Master Equation [6,7]. This model is utilized to describe the dynamics of systems for which molecular populations of some species are low or noise is significant. It assumes that the system state is a Markov process [6]. It is generally impracticable to solve this model analytically, except for very simple systems.

Gillespie developed the Stochastic Simulation Algorithm (SSA) [8,9], a Monte Carlo technique for simulating statistically exact realizations of the stochastic process whose distribution is governed by the Chemical Master Equation. The random time change representation of the stochastic process depicting the system state was introduced in [10]. Based on this representation, Rathinam et al. [11] designed an exact Monte Carlo method for the Chemical Master Equation, the Random Time Change algorithm. Other simulation strategies for stochastic models of biochemically reacting systems were presented in the literature (for references see, e.g., [12,13,14,15]).

The biochemical networks arising in applications may be quite complex, involving many reactions and/or species, which means that their mathematical models have many parameters. Some of the values of a model’s kinetic parameters may not be known [16,17] and they may need to be estimated from the available data. Also, certain parameters have a substantial influence on the system’s output. Thus, it is essential to study the system’s behavior when these parameters are perturbed. While stochastic discrete models of biochemical systems capture the inherent randomness observed in cellular processes, they pose challenges with regard to their parameter estimation and identification. Hence, developing efficient and accurate methods for identifying and estimating their parameters would be a key advance in studying these models.

Practical identifiability (or estimability) analysis aims to establish if the parameters can be accurately and reliably estimated from the available data [18]. In this context, identifiable parameters are those which can be determined with high confidence from the observed behavior of the system; otherwise, the parameters are unidentifiable. Using practical identifiability, one can select subsets of parameters that significantly impact the behavior of the system. If the parameters in such a subset are not interdependent, then they are identifiable. These parameters can be accurately estimated when sufficient and quality data is available, and their accurate estimation is crucial for building the model. Also, these parameters may provide insight into the key underlying mechanisms of the biochemical system. Furthermore, the identifiability analysis helps select the unidentifiable parameters, which have a negligible impact on the model behavior and can be eliminated, thus guiding model reduction. There exist numerous studies of identifiability analysis for deterministic models, such as the reaction rate equations [19,20,21,22,23,24,25,26]. Nonetheless, much less work has been dedicated to parameter estimability of stochastic models of biological processes.

One important method for practical identifiability is to utilize sensitivity analysis. Local sensitivity analysis assesses the change in the system’s behavior caused by a small variation in the value of a certain parameter. Insignificant changes in the system dynamics indicate that the specific parameter is not important, and thus it is not identifiable. Also, a parameter is not identifiable if it is correlated with other parameters, such that a variation in its value can be compensated by suitable adjustments in other parameters. For stochastic models, finite-difference methods can be used to estimate the sensitivity of the expected value of the given function of the system state. In the class of finite-difference sensitivity estimators for the Chemical Master Equation, those employing exact Monte Carlo simulation methods are the Coupled Finite Difference method of Anderson [27], the Common Reaction Path scheme (based on the Random Time Change algorithm) and the Common Random Number strategy (utilizing the SSA) of Rathinam et al. [11]. These estimators utilize coupled perturbed and unperturbed trajectories to approximate sensitivities. The coupling lowers the variance of the estimator so that the method requires fewer realizations to achieve the same accuracy of the estimation. Due to this, the computational time of the algorithm is reduced, for a prescribed accuracy. Of the three strategies, the Coupled Finite Difference algorithm has the lowest variance of the estimator [28]. These schemes perform best for non-stiff models. For stiff problems, finite-difference techniques can be applied with various coupled tau-leaping strategies to increase the efficiency of the simulation [29].

In this work, we consider the problem of practical parameter identifiability for stochastic discrete biochemical networks modeled with the Chemical Master Equation. This is a critical problem, and a direct extension of the techniques developed for ordinary differential equations to stochastic discrete models is not possible. Our contribution is generalizing a method by Gábor et al. [30] to find the highest parameter identifiable sets for models of biochemical systems, from the continuous deterministic to the stochastic discrete models of well-stirred biochemical systems, which is a difficult task. The proposed method identifies the subsets of parameters that are independent and significant for the model’s behavior, based on the existing data, and thus are identifiable. We utilize local sensitivity estimations to study parameter estimability. For approximating sensitivities, we apply finite-difference techniques, namely the Coupled Finite Difference [27], the Common Reaction Path, and the Common Random Number methods [11]. We make use of the normalized sensitivity matrix to develop several identifiability metrics, which adapt existing techniques for the reaction rate equations [19,20] to the more challenging Chemical Master Equation model. In addition, we apply the singular value decomposition of the non-dimensional sensitivity matrix, to determine its rank. This analysis helps gain insight into the interrelations between parameters. Furthermore, the proposed methodology can be employed to decide which parameters can be reliably estimated from the available data, for the Chemical Master Equation, and may assist experimental design for more accurate parameter approximations. It is worth noting that, in general, the expected value of the system state governed by the Chemical Master Equation may not satisfy the deterministic reaction rate equations, when some reactions are of second or higher order [14].

This paper is structured as follows. Section 2 is dedicated to the background on stochastic discrete models for well-stirred biochemical networks and their simulation methods, parametric sensitivity schemes for stochastic and deterministic models, and practical identifiability techniques, including the new algorithm for selecting subsets of identifiable parameters. The proposed algorithm is tested on various stochastic models arising in applications in Section 3. Section 4 presents a summary of our results.

## 2. Materials and Methods

### 2.1. Background

Suppose a system has *N* biochemical species, denoted by $S_{1},S_{2},...,S_{N}$, that undergo *M* distinct chemical reactions. It is maintained at a constant temperature, in a constant volume. Provided that the biochemical network is well-stirred, it may be represented by a state vector,

$$X\left(t\right)={\left[X_{1}\left(t\right),X_{2}\left(t\right),...,X_{N}\left(t\right)\right]}^{T},$$

where X(t) has entries $X_{i}\left(t\right)$, the amount of $S_{i}$ molecules in the system at time *t*. A reaction $R_{j}$ produces a variation in the system state, which is given by the state change vector $\nu_{j}\in R^{N}$,

$$\nu_{j}={\left[\nu_{1j},\nu_{2j},...,\nu_{Nj}\right]}^{T}\;,$$

where $\nu_{ij}$ is the perturbation in the molecular amount of $S_{i}$ after the reaction fires. If one reaction $R_{j}$ happens during the time interval [t,t+Δt], then the resulting state is $X\left(t+\Delta t\right)=X\left(t\right)+\nu_{j}$. The array having $\nu_{j}$ as the *j*-th column is called the stoichiometric matrix. Also associated with the reaction $R_{j}$, we can define the propensity function $a_{j}$, by $a_{j}\left(x\right)dt=$ the probability that a single reaction $R_{j}$ occurs between [t,t+dt), assuming that the system state at time *t* is *x*. The form of the propensity function $a_{j}$ is determined by the type of reaction. For a first-order reaction, $S_{m}\overset{c_{j}}{\to }products$, the propensity is expressed as $a_{j}\left(X\left(t\right)\right)=c_{j}X_{m}\left(t\right)$. For a second-order reaction, $S_{m}+S_{n}\overset{c_{j}}{\to }products$, the propensity is $a_{j}\left(X\left(t\right)\right)=c_{j}X_{m}\left(t\right)X_{n}\left(t\right)$, if m≠n and $a_{j}\left(X\left(t\right)\right)=\frac{1}{2}c_{j}X_{m}\left(t\right)\left(X_{m}\left(t\right)-1\right)$, if m=n.

#### 2.1.1. Chemical Master Equation

To study the behavior of the well-stirred biochemical system, we need to determine $P(x,t|x_{0},t_{0})$, the probability of the system state being X(t)=x at time *t*, if at $t_{0}$ it was $X\left(t_{0}\right)=x_{0}$. This probability satisfies the Chemical Master Equation [6,7]

(1) $$\frac{d}{dt}P\left(x,t|x_{0},t_{0}\right)=\sum_{j=1}^{M}\left[a_{j}\left(x-\nu_{j}\right)P\left(x-\nu_{j},t|x_{0},t_{0}\right)-a_{j}\left(x\right)P\left(x,t|x_{0},t_{0}\right)\right]\;.$$

This is a stochastic discrete model. It is a linear system of ordinary differential equations, each equation describing the probability of the system being in a particular state *x*. The biochemical system state X(t) is a discrete in space and continuous in time Markov process. The space of all possible states is typically quite large, and in such cases the Chemical Master Equation is of very high dimension. Therefore, it is challenging to solve it directly, except for some simple systems.

As an alternative to solving the Chemical Master Equation directly, it is possible to generate correct trajectories one by one. Gillespie [8,9] proposed a Monte Carlo strategy to compute such trajectories, which are in exact agreement with the probability distribution associated with the discrete stochastic model (1). The strategy, also referred to as the Stochastic Simulation Algorithm (SSA), has been broadly employed for solving stochastic models in Systems Biology [3,14,31]. The SSA is described below.

##### Gillespie’s Algorithm

1. Initialize the time $t\leftarrow t_{0}$ and the state of the system, $X\left(t\right)\leftarrow x_{0}$.
2. While t<T
3. Calculate each propensity $a_{j}\left(X\left(t\right)\right)$ for j=1,…,M and the sum $a_{0}\left(X\left(t\right)\right)\leftarrow \sum_{r=1}^{M}a_{r}\left(X\left(t\right)\right)$
4. Sample two uniform random variables over [0,1], to obtain $\eta_{1}$, $\eta_{2}$.
5. Evaluate the time τ and the index *j* of the next occurring reaction, according to
  (a) $\tau \leftarrow -\left(\ln\eta_{1}\right)/a_{0}\left(x\right)$
  (b) j← the smallest integer fulfilling $\sum_{r=1}^{j}a_{r}\left(x\right)>\eta_{2}a_{0}\left(x\right)$
6. Update the state $X\left(t+\tau \right)\leftarrow X\left(t\right)+\nu_{j}$ and the time t←t+τ.
7. End while.

The Random Time Change (RTC) algorithm [11], based on the Random Time Change representation [10], is another exact Monte Carlo simulation strategy for the Chemical Master Equation. We refer the reader to [11] for details on this algorithm.

#### 2.1.2. Chemical Langevin Equation

An intermediate model between the Chemical Master Equation and the reaction rate equation is the Chemical Langevin Equation [32]. This is a system of stochastic differential equations of size equal to the number of reacting species. The Chemical Langevin Equation is a reduction in the Chemical Master Equation model assuming that the biochemical system has a macroscopically infinitesimal scale in time step such that, over δt, every reaction occurs multiple times and, at the same time, its propensity function does not vary significantly. Under these assumptions, the system state is governed by

(2) $$dX\left(t,c\right)=\sum_{j=1}^{M}\nu_{j}a_{j}\left(X\left(t,c\right),c\right)dt+\sum_{j=1}^{M}\nu_{j}\sqrt{a_{j}\left(X\left(t,c\right),c\right)}dW_{j}\left(t\right)$$

where $W_{j}$ are independent Wiener processes for j=1,…,M. The state X(t) may be approximated by a Markov process continuous in space. Equation (2) represents the Chemical Langevin Equation.

#### 2.1.3. Reaction Rate Equation

A coarser level of resolution in modeling biochemically reacting networks is provided by the continuous deterministic model of chemical kinetics. This model, known as the reaction rate equations, is valid under the assumption of the thermodynamic limit. In the thermodynamic limit, the molecular amounts for all species and the system volume tend towards infinity, as the concentrations of species within the system remain constant. Hence, the stochastic terms in the Chemical Langevin Equation are much smaller than the deterministic terms. As a result, the Chemical Langevin Equation model reduces to the reaction rate equations, in the thermodynamic limit. This condition is satisfied when all $S_{i}$ molecular counts are very large. The reaction rate equations (RRE) are of the form

(3) $$\frac{dX(t,c)}{dt}=\sum_{j=1}^{M}\nu_{j}a_{j}\left(X\left(t,c\right),c\right)\;.$$

Equation (3) is a set of ordinary differential equations, with one equation for each biochemical species. In the event that all reactions in the system are of order at most one, the reaction rate equation can be obtained from the Chemical Master Equation (1), by considering the expected value of the system state. However, in general, the evolution of the mean trajectory in the Chemical Master Equation model does not obey the continuous deterministic model. Then, the RRE does not properly depict the true behavior of the biochemical network. In fact, there are numerous cellular networks for which noise significantly influences the system dynamics [12,31,33].

### 2.2. Parametric Correlations

Sensitivity analysis plays a central role in constructing models [24]. It assesses how changes in parameters cause variations in a model’s output. If a negligible adjustment in a parameter leads to significant alterations in the outcomes, we consider the model to be sensitive to that specific parameter. Precise estimations are not necessary for parameters with low sensitivity. Conversely, parameters with high associated sensitivity become key control points for the behavior of the system. In what follows, we shall focus on the sensitivity analysis of system outputs with respect to rate parameters.

#### 2.2.1. Parametric Sensitivity for the Chemical Master Equation

Let *f* be a function of interest of the system state and *c* a model parameter. In the stochastic setting, the local sensitivity with respect to a parameter *c* is defined as $\frac{\partial }{\partial c}E\left[f\left(X\left(t,c\right)\right)\right]$ where E(·) is the expected value. Popular methods for estimating local sensitivities with respect to the model’s parameters for the Chemical Master Equation often rely on finite-difference schemes and Monte Carlo methods for generating the perturbed and unperturbed trajectories. By forward finite-difference schemes, one can estimate $\frac{\partial }{\partial c}E\left[f\left(X\left(t,c\right)\right)\right]\approx \left\{E\left[f\left(X\left(t,c+\theta \right)\right)\right]-E\left[f\left(X\left(t,c\right)\right)\right]\right\}/\theta$, where θ is a small perturbation of the parameter of interest, *c*. To efficiently approximate the sensitivity by Monte Carlo methods, the trajectories for X(t,c+θ) and X(t,c) are generated using common random numbers. Among such methods are the Common Random Number (CRN), the Common Reaction Path (CRP) algorithms [11], and the Coupled Finite-Difference (CFD) algorithm [27].

#### 2.2.2. Common Random Number

The Common Random Number presented in [11] is a finite-difference numerical method for estimating parametric sensitivities for the stochastic discrete model (1). It reuses random numbers to generate the perturbed and unperturbed paths. In doing so, it reduces the variance of the sensitivity estimator, and thus it has increased efficiency compared to a strategy based on independent random numbers. For the *r*-th iteration, it computes two SSA trajectories, $X_{\left[r\right]}\left(t,c+\theta \right)$ -the perturbed and $X_{\left[r\right]}\left(t,c\right)$ -the unperturbed path, each employing the same stream of uniform (0,1) random numbers. Usually, the coupling of the CRN technique is less efficient than that of the CRN and CFD schemes [27]. The sensitivity of the *r*-th path is approximated by

(4) $$Z_{\left[r\right]}\left(t,c\right)=\frac{f\left(X_{\left[r\right]}\left(t,c+\theta \right)\right)-f\left(X_{\left[r\right]}\left(t,c\right)\right)}{\theta }\;,$$

while an estimate of the sensitivity is obtained from the sample mean $(\sum_{i=1}^{R}Z_{\left[r\right]}\left(t,c\right))/R$, *R* being the number of paired trajectories simulated.

#### 2.2.3. Common Reaction Path

The Common Reaction Path technique is also a finite-difference sensitivity estimator for the Chemical Master Equation [11]. The CRP strategy applies the RTC algorithm to simulate sample paths. In this method, coupling of the processes involves some independent unit-rate Poisson processes, ${\left\{Y_{j}\right\}}_{1\leq j\leq M}$. The coupling of the perturbed—X(·,c+θ) and unperturbed—X(·,c) processes is achieved using the random time change representation

(5) $$\begin{array}{c} \begin{array}{c} X\left(t,c\right)=x_{0}+\sum_{j=1}^{M}\nu_{j}Y_{j}\left(\int_{0}^{t}a_{j}\left(X\left(s,c\right),c\right)ds\right)\\ X\left(t,c+\theta \right)=x_{0}+\sum_{j=1}^{M}\nu_{j}Y_{j}\left(\int_{0}^{t}a_{j}\left(X\left(s,c+\theta \right),c+\theta \right)ds\right) \end{array} \end{array}$$

The *r*-th iteration of the CRP algorithm generates the paired trajectories $X_{\left[r\right]}\left(t,c+\theta \right)$ and $X_{\left[r\right]}\left(t,c\right)$ with the RTC algorithm, each using the same *M* independent streams of unit-rate exponential random numbers. As before, the sensitivity of the *r*-th trajectory is estimated by (4). This coupling has been shown to be typically stronger than that of the CRN method, leading to a lower variance of the estimation [11,27].

#### 2.2.4. Coupled Finite-Difference

Another finite-difference sensitivity estimator for the stochastic discrete model is the Coupled Finite-Difference scheme [27]. The CFD method relies on the random time change representation of the unperturbed and perturbed processes

(6) $$\begin{array}{ccc} X(t,c) & = & x_{0}+\sum_{j=1}^{M}\nu_{j}Y_{j}^{\left(1\right)}\left(\int_{0}^{t}\min\left(a_{j}\left(X\left(s,c\right),c\right),a_{j}\left(X\left(s,c+\theta \right),c+\theta \right)\right)ds\right)\\ \; & + & \sum_{j=1}^{M}\nu_{j}Y_{j}^{\left(2\right)}\left(\int_{0}^{t}[a_{j}\left(X\left(s,c\right),c\right)-\min\left(a_{j}\left(X\left(s,c\right),c\right),a_{j}\left(X\left(s,c+\theta \right),c+\theta \right)\right]ds\right)\\ X(t,c+\theta ) & = & x_{0}+\sum_{j=1}^{M}\nu_{j}Y_{j}^{\left(1\right)}\left(\int_{0}^{t}[\min\left(a_{j}\left(X\left(s,c\right),c\right),a_{j}\left(X\left(s,c+\theta \right),c+\theta \right)\right]ds\right)\\ \; & + & \sum_{j=1}^{M}\nu_{j}Y_{j}^{\left(3\right)}\left(\int_{0}^{t}[a_{j}\left(X\left(s,c+\theta \right),c+\theta \right)-\min\left(a_{j}\left(X\left(s,c\right),c\right),a_{j}\left(X\left(s,c+\theta \right),c+\theta \right)\right]ds\right) \end{array}$$

where ${\left\{Y_{j}^{\left(1\right)}\right\}}_{1\leq j\leq M}$, ${\left\{Y_{j}^{\left(2\right)}\right\}}_{1\leq j\leq M}$. and ${\left\{Y_{j}^{\left(3\right)}\right\}}_{1\leq j\leq M}$ are independent unit-rate Poisson processes. Furthermore, the CFD strategy uses a version of the Next Reaction Method to compute the coupled perturbed and unperturbed trajectories, $X_{\left[r\right]}\left(t,c+\theta \right)$ and $X_{\left[r\right]}\left(t,c\right)$, and (4) to approximate the local sensitivity of the *r*-th path. Among the finite-difference sensitivity estimators with exact underlying simulation techniques for the CME, the CFD performs the best, followed by the CRP and the CRN [27,28]. Indeed, the CFD achieves the smallest variance of the sensitivity estimator of the three methods described above [28]. As a consequence, for the same number of trajectories simulated, we shall consider in our investigations the CFD sensitivity approximations to be the most accurate and reliable.

#### 2.2.5. Parametric Sensitivity for the Chemical Langevin Equations

Glasserman [34] developed a technique for computing pathwise parametric sensitivities for certain problems modeled by stochastic differential equations. This method was applied to the Chemical Langevin Equation (CLE) model in [33]. For computing the sensitivity of each path, we differentiate Equation (2) with respect to parameter *c* and obtain

(7) $$\begin{array}{c} d(\frac{\partial X}{\partial c})=\sum_{j=1}^{M}\nu_{j}[\frac{\partial a_{j}\left(X\right)}{\partial X}\frac{\partial X}{\partial c}+\frac{\partial a_{j}\left(X\right)}{\partial c}]\left(t\right)dt\\ +\sum_{j=1}^{M}\nu_{j}\left[\frac{1}{2\sqrt{a_{j}\left(X\right)}}\left(\frac{\partial a_{j}\left(X\right)}{\partial X}\frac{\partial X}{\partial c}+\frac{\partial a_{j}\left(X\right)}{\partial c}\right)\right]\left(t\right)dW_{j}\;. \end{array}$$

Solving the coupled system of Equations (2) and (7) for (X,∂X/∂c) will determine the pathwise sensitivities. At time t=0, the local sensitivities with respect to the rate parameters are zero. The Chemical Langevin Equation is, in general, valid when all molecular amounts are sufficiently large. Effective simulation strategies for this model require adaptive time-stepping methods [35,36].

#### 2.2.6. Parametric Sensitivity for the Reaction Rate Equations

In the deterministic scenario, the behavior of the biochemical system is governed by the reaction rate Equation (3). To find the local sensitivity for this model, the derivative with respect to the desired kinetic parameter is applied to Equation (3), yielding

(8) $$\frac{d}{dt}S=\sum_{j=1}^{M}\nu_{j}\left(\frac{\partial a_{j}\left(X\left(t,c\right),c\right)}{\partial c}+\sum_{i=1}^{N}\frac{\partial a_{j}\left(X\left(t,c\right),c\right)}{\partial X_{i}}S_{i}\right)\;.$$

Here, S=∂X(t,c)/∂c is the sensitivity with respect to parameter *c*. The sensitivity is computed by solving for (X,S) the system of ordinary differential Equations (3) and (8), with the initial conditions $X\left(0,c\right)=x_{0}$ and S(0)=0. The deterministic model is applicable when all reacting molecular populations are very large. Nonetheless, when low molecular counts of some species exist or noise plays a significant role, this approach may fail in accurately capturing the characteristics of the biochemical system. Then, deterministic techniques for sensitivity-based identifiability analysis are not valid.

### 2.3. Practical Identifiability Analysis

When a model’s performance is investigated, it is important to evaluate the accuracy of the parameter values. Still, poor or noisy data, interdependence of parameters, or weak dependence of the system dynamics on certain parameters may hinder the accurate estimation of parameter values. As a result, it is possible for these values to change significantly, without influencing the model’s output. Consequently, the concept of identifiability is essential for the analysis of a mathematical model [19,24].

Identifiability can be classified into two main categories: structural identifiability and practical identifiability. For a structurally identifiable model, there exists a unique parameterization for any specified output of the model (see, e.g.,  [21,26]). On the other hand, practical identifiability involves detecting non-identifiable parameters by fitting the model to data that closely resemble the available observations (see, e.g., [18,19,22,25] for analyses of deterministic models). For this type of identifiability, it is helpful to study the parametric sensitivity of the model. In this work, we use sensitivity-based identifiability for the Chemical Master Equation. We determine identifiability and collinearity indexes by generalizing methods for deterministic models [19] to the more challenging case of stochastic discrete biochemical systems.

#### 2.3.1. Sensitivity-Based Identifiability Analysis

Several identifiability strategies for deterministic models exist in the literature. One such approach by Brun et al. [19] is based on local sensitivity analysis of deterministic models. Sensitivity analysis quantifies the impact of parameter variations on the system’s dynamics.

Below, we review some techniques for identifiability analysis of deterministic models relying on local parametric sensitivity. These techniques can be applied to the reaction rate Equation (3). Denote by

(9) $$S_{ik}\left(X,t,c\right)=\frac{\partial X_{i}\left(t,c\right)}{\partial c_{k}}$$

the local sensitivity of the molecular amount $X_{i}\left(t,c\right)$ at time *t*, with respect to the kinetic parameter $c_{k}$. For time *t*, the parametric sensitivity matrix is $S\left(X,t,c\right)=\frac{\partial }{\partial c}X\left(t,c\right)={\left\{S_{ik}\left(X,t,c\right)\right\}}_{1\leq i\leq N,1\leq k\leq M}$. In addition, the non-dimensional sensitivity coefficient corresponding to the *i*-th species and the parameter $c_{k}$ at time *t* is

(10) $$s_{ik}\left(t\right)=\frac{c_{k}}{X_{i}\left(t,c\right)}\frac{\partial X_{i}\left(t,c\right)}{\partial c_{k}}\;.$$

Here, $c=[c_{1},\ldots ,c_{M}]$ is the vector of kinetic parameters associated to reactions ${\left\{R_{j}\right\}}_{1\leq j\leq M}$. Furthermore, let $t_{1}<t_{2}<...<t_{L}$ be a sequence of time-points spanning the integration interval [0,T]. Ideally, some of these time-points should be inside the interval corresponding to the biochemical network’s transient behavior, when applicable. Also, consider the concatenated non-dimensional sensitivity matrix, for all the time-points in the grid, and apply the normalization (10) for each entry,

(11) $$s\left(X,c\right)=\left(\begin{array}{ccc} s_{11}\left(t_{1}\right) & \cdots & s_{1M}\left(t_{1}\right)\\ \vdots & \ddots & \vdots \\ s_{N1}\left(t_{L}\right) & \cdots & s_{NM}\left(t_{L}\right) \end{array}\right)\;.$$

To rank the parameters of the model, we utilize the non-dimensional sensitivity matrix of size (NL)×M from (11). The *k*-th column in this matrix measures the sensitivities with respect to $c_{k}$, the rate parameter of reaction $R_{k}$. Let us calculate the norm of each column in the sensitivity matrix (11) to obtain a parameter ranking. The norm of each column $s_{k}\left(X,c\right)={\left[s_{1k}\left(t_{1}\right),\ldots ,s_{Nk}\left(t_{1}\right),\ldots ,s_{1k}\left(t_{L}\right),\ldots ,s_{Nk}\left(t_{L}\right)\right]}^{T}$ serves as a measure of the significance of parameter $c_{k}$ on the dynamics of the system. A higher norm indicates that altering that parameter value has a substantial impact on the system state. Parameters can be arranged in order of their significance. The following sensitivity measure is employed for evaluating the significance of the parameters, based on the sensitivity matrix (adapted after [19])

(12) $$\delta_{k}^{msqr}=\sqrt{\frac{1}{n}\sum_{i=1}^{n}s_{ik}^{2}}\;.$$

The larger the measure $\delta_{k}^{msqr}$, the more significant the parameter $c_{k}$ is (for 1≤k≤M).

#### 2.3.2. Parameter Collinearity

Extensive research has been conducted to examine the collinearity in various problems. Brun et al. [19] introduces a strategy for identifying parameter relationships based on collinearity analysis, in the deterministic framework, and presents a novel approach to explore the connections between parameters. Note that the columns of a matrix *B* are nearly linearly dependent (or near collinear) if a non-zero vector $z={\left[z_{1},...,z_{M}\right]}^{T}$ exists such that Bz≈0, where *B* has *M* columns. If the Bz=0 holds and z≠0, the columns of *B* are linearly dependent (or collinear).

Now, take the normalized sensitivity matrix $\tilde{S}$, having as the *m*-th column the vector

$${\tilde{s}}_{m}\left(X,c\right)=\frac{s_{m}\left(X,c\right)}{∥s_{m}{\left(X,c\right)∥}_{2}}\;,$$

for 1≤m≤M. It is useful to first normalize these vectors, to prevent biases due to differences in the absolute value of local sensitivities for various parameters. A large norm of $∥s_{m}{∥}_{2}$ indicates that a small variation in parameter $c_{m}$ can significantly impact the system’s behavior; thus, this parameter is important. For this parameter to be identifiable, it should not be correlated with other parameters.

Let us consider any subsets *K* of *k* parameters (k≤M) from the set of parameters $\left\{c_{1},c_{2},\ldots ,c_{M}\right\}$ and the corresponding sub-matrix ${\tilde{S}}_{K}\left(X,c\right)$ of the normalized sensitivity matrix, with columns the *k* sensitivity vectors. A measure of collinearity of the subset *K* of parameters, with corresponding matrix ${\tilde{S}}_{K}$, is given by

(13) $$CI_{K}=\frac{1}{\min_{{∥z∥}_{2}=1}{∥{\tilde{S}}_{K}z∥}_{2}}=\frac{1}{\sqrt{\lambda_{k}}}$$

where $\lambda_{k}$ is the minimum eigenvalue of the matrix ${\tilde{S}}_{K}^{T}{\tilde{S}}_{K}$ and ${∥\cdot ∥}_{2}$ is the norm-2 of a vector. The measure (13) is known as the collinearity index of the subset *K* [19,30]. The closest the columns of the matrix ${\tilde{S}}_{K}$ are to a linearly dependent set of vectors, the smallest $\min_{{∥z∥}_{2}=1}{∥{\tilde{S}}_{K}z∥}_{2}$ is. Thus, a large collinearity index $CI_{K}$ indicates a high level of collinearity of the parameters in the set. This implies that changes in the model dynamics due to small perturbations in one of the parameters of the almost collinear set may be prevented by suitable variations in the other parameters of the set. As a consequence, if a set of parameters is collinear, it is not identifiable. According to [19], a subset of parameters is considered identifiable if the associated collinearity index satisfies $CI_{K}<20$. With this observation, it is possible to uncover the subsets of model parameters that can be identified as well as those that cannot be identified. The collinearity index may be computed for all the subsets *K* of the parameter space, to determine the parameter subsets that are not collinear. When a group of parameters has a high collinearity index, any set containing it as a subset will also have a high collinearity index.

Another technique to assess the identifiability of the model parameters is to use the singular value decomposition (SVD) of a matrix. In general, the SVD [37,38] of an n×M matrix *s* is

(14) $$s=U\Sigma V^{T}\;,$$

where the U is an n×n unitary matrix, V is an M×M unitary matrix and Σ is an n×M non-negative diagonal matrix with the diagonal entries

$$\sigma_{1}\geq \sigma_{2}\geq \ldots \sigma_{r}>\sigma_{r+1}=\cdots =\sigma_{M}=0\;.$$

The values ${\left\{\sigma_{m}^{2}\right\}}_{1\leq m\leq M}$ are the eigenvalues of the matrix $s^{T}s$. The index *r* measures the rank of the matrix *s* and it is the largest number of linearly independent columns of this matrix. Numerically, the singular values $\sigma_{r+1},\cdots ,\sigma_{M}$, which are below a specified small tolerance are considered practically zero. In this work, we use the singular value decomposition of the matrix *s* to determine its rank. This rank is a reliable measure of the number of rate parameters that are not collinear. Furthermore, zero or very close to zero singular values show that the group of all the reaction rate parameters of the model are collinear. Therefore, there are some model parameters that cannot be estimated from the available data.

Brun et al. [20] also introduced a determinant measure

(15) $$\rho_{k}=\det{\left(S_{K}^{T}S_{K}\right)}^{1/2k}$$

to find the appropriate number of parameters to estimate.

The metrics considered above can be utilized to determine the identifiability of parameter sets as follows. The sensitivity measure $\delta_{k}^{msqr}$ is used to evaluate the importance of each parameter $c_{k}$. On the other hand, the collinearity index measures whether the set *K* of parameters are independent, whenever $CI_{K}<20$. In the case that both conditions are satisfied, (a) the parameters in the subset *K* are not collinear and (b) each parameter in the group is important, the parameters in *K* are identifiable. Finally, the determinant $\rho_{K}$ can be employed to compare the identifiability of various groups of parameters.

#### 2.3.3. Method for Selecting Subsets of Identifiable Parameters

The practical identifiability methods presented above were developed for continuous deterministic models [19,20], and are thus applicable for the reaction rate equation model. However, this model may fail to faithfully represent the behavior of biochemical systems, which involve low molecular counts of some species. Consequently, new methodologies are required for the parameter identifiability of stochastic discrete models of biochemical systems. In this work, we develop novel strategies for determining sets of identifiable parameters for the Chemical Master Equation. We generalize the work of Gábor et al. [30] on identifying subsets of identifiable parameters in deterministic models, to address the much more challenging case of stochastic discrete models of well-stirred biochemical systems. This generalization is essential as stochasticity plays a significant role in accurately modeling real-world biological systems, and our approach allows for an in-depth study of more complex biochemical networks encountered in applications.

The measures presented above were designed for deterministic models. We aim to adapt these measures to systems modeled by the Chemical Master Equation. For this model, the sensitivity coefficients are computed as

$$S_{ik}\left(E\left[X\right],t\right)=\frac{\partial }{\partial c_{k}}E\left[X_{i}\left(t,c\right)\right].$$

Then, we shall compute the sensitivity matrix for the CME according to

(16) $$S\left(t\right)=\frac{\partial E[X(t,c)]}{\partial c}=\left(\begin{array}{ccc} \frac{\partial }{\partial c_{1}}E\left(X_{1}\left(t,c\right)\right) & \cdots & \frac{\partial }{\partial c_{M}}E\left(X_{1}\left(t,c\right)\right)\\ \vdots & \ddots & \vdots \\ \frac{\partial }{\partial c_{1}}E\left(X_{N}\left(t,c\right)\right) & \cdots & \frac{\partial }{\partial c_{M}}E\left(X_{N}\left(t,c\right)\right) \end{array}\right)\;.$$

Take a sequence of time-points $0=t_{1}<t_{2}<\ldots <t_{L}=T$, relevant to the biochemical system under consideration. The fully normalized (non-dimensional) sensitivity coefficient of the *i*-th species with respect to the $c_{k}$ parameter at time $t_{\ell }$ is

(17) $$s_{ik}\left(t_{\ell }\right)=\frac{c_{k}}{E[X_{i}\left(t_{\ell },c\right)]}\frac{\partial }{\partial c_{k}}E\left[X_{i}\left(t_{\ell },c\right)\right]\;\mathrm{for}\;1\leq i\leq N,\;1\leq k\leq M.$$

The concatenated non-dimensional sensitivity matrix over these discrete time-points with entries (17) is

(18) $$s\left(E\left[X\right],c\right)=\left(\begin{array}{ccc} s_{11}\left(t_{1}\right) & \cdots & s_{1M}\left(t_{1}\right)\\ \vdots & \ddots & \vdots \\ s_{N1}\left(t_{L}\right) & \cdots & s_{NM}\left(t_{L}\right) \end{array}\right)\;.$$

Normalizing the *ℓ*-th column of matrix (18), namely $s_{\ell }\left(E\left[X\right],c\right)$, gives

(19) $${\tilde{s}}_{\ell }\left(E\left[X\right],c\right)=\frac{s_{\ell }\left(E\left[X\right],c\right)}{∥s_{\ell }{\left(E\left[X\right],c\right)∥}_{2}}\;.$$

Finally, the normalized sensitivity matrix $\tilde{S}$ has ${\tilde{s}}_{\ell }\left(E\left[X\right],c\right)$ as it is *ℓ*-th column. For the Chemical Master Equation, the sensitivity measure $\delta_{k}^{msqr}$ and the collinearity index $CI_{K}$ are computed using (12) and (13), respectively, for the sensitivity matrix of the expected value E[X] rather than the system state *X*, as was the case for the reaction rate equation.

Moreover, we will employ the finite-difference methods described above to estimate parametric sensitivities. Recall that a finite-difference estimate of the sensitivity with respect to parameter $c_{k}$, over *R* coupled perturbed and unperturbed paths, is

$$\frac{\partial }{\partial c_{k}}E\left[X\left(t,c\right)\right]\approx Z_{R}=\frac{1}{R}\sum_{r=1}^{R}\frac{X_{\left[r\right]}\left(t,c_{k}+\theta \right)-X_{\left[r\right]}\left(t,c_{k}\right)}{\theta }\;.$$

While we compute the coupled trajectories using the CFD, CRP, or CRN strategies, our method can be applied to other finite-difference sensitivity estimators [29].

The measure (12) can be calculated to rank parameters from most to least influential. Small values of $\delta^{msqr}$ correspond to parameters with a small influence on the model. We select those parameters that show the value of $\delta^{msqr}$ larger than 0.2 [39]. With an initial ranked list, we compute the collinearity indices for this list. This method can be applied to models of moderate size.

Algorithm 1 calculates the normalized sensitivity matrix, as follows. A grid with *L* time-points ranging from 0 to *T* is selected. We choose equally distributed time steps, such that data is collected from all important regions of the interval of integration. This depends on the particular model. We note that an adaptive time-stepping procedure can be included instead. Then, the sensitivity matrices $S(t_{l})$ from Equation (16) are approximated with a specific finite-difference sensitivity estimator. Afterwards, we compute the concatenated non-dimensional sensitivity matrix *s*. We normalize each column of *s* individually to ensure consistency and comparability. The normalization implies dividing each column $s_{k}$ by its vectorial norm-2. Column normalization yields a matrix denoted by $\tilde{S}$. This matrix has as its *k*-th column $\left\{{\tilde{s}}_{k}\right\}=s_{k}/{∥s_{k}∥}_{2}$. Also, for each parameter $c_{k}$ we compute the sensitivity measure $\delta_{k}^{\mathrm{msqr}}$ from Equation (12), using the entries of the *k*-th columns of the sensitivity matrices $S(t_{\ell })$.
**Algorithm 1** Computing the Normalized Sensitivity Matrix**Initialize**: Time grid: $0=t_{1}<t_{2}<\ldots <t_{L}=T$.**Input**: Estimates of sensitivity matrices $S(t_{\ell })$ from (16).Compute the concatenated non-dimensional sensitivity matrix *s* from (18) with entries (17)**for** k=1 to *M* **do**    normalize ${\tilde{s}}_{k}=\frac{s_{k}}{∥s_{k}{∥}_{2}}$ where $s_{k}$ is the *k*-th column of *s* and ${∥\cdot ∥}_{2}$ is norm-2**end for**Compute normalized matrix $\tilde{S}={\left\{{\tilde{s}}_{k}\right\}}_{1\leq k\leq M}$**for** k=1 to *M* **do**    Compute sensitivity measure $\delta_{k}^{msqr}$ according to (12) for parameter $c_{k}$**end for**

In Algorithm 2, we introduce a method for the selection of identifiable parameter subsets based on sensitivity measures and collinearity indices. This procedure extends and refines a methodology by Gábor et al. [30] from the deterministic to the more difficult case of stochastic biochemical networks. The goal of Algorithm 2 is to iteratively assess the practical identifiability of subsets of model parameters. A threshold value is set for the collinearity indices, which measure the level of collinearity between parameter groups. The threshold value determines the acceptable level of collinearity. With a normalized sensitivity matrix obtained from Algorithm 1 as input, the following steps are considered. The parameters are ranked according to their sensitivity measure, those with a sensitivity measure below a critical value (chosen here as 0.2) are considered unimportant and may be discarded. If the ranked list of parameters is of moderate size, combinations of parameters are generated. For each combination, the algorithm computes the corresponding collinearity index. This involves calculating the collinearity indices for pairs, triples, etc. These indices quantify the degree of collinearity between the parameters of a certain group. When the computed collinearity index for a parameter subset is below the threshold value, that subset of parameters is deemed identifiable. By applying this algorithm, a subset of parameters with low collinearity and high identifiability can be selected. This allows for the reduction in model complexity and for the accurate and reliable estimation of the most important parameters, from the input data.
**Algorithm 2** Selecting a Subset of Identifiable Parameters**Input**: Normalized sensitivity matrix;**Input**: Set threshold value of collinearity index: $CI_{cr}=20$**Require**: Rank parameters $c_{j}$ based on $\delta_{j}^{msqr}>0.2$**if** Ranked list is of moderate size **then**    1: Number of all combinations: C=Length(combnk)    2: Compute collinearity indices for all combinations of the ranked list of parameters:    **for** k = 1 to C **do**        For every combination of the ranked list of parameters, calculate the collinearity indices:        $CI_{2}=collinearityindex\left(pairs\right)$, $CI_{3}=collinearity\left(triples\right)$, etc.        $L_{2}=pair\;combination$, $L_{3}=triple\;combination$, etc.    **end for****end if****if** $CI_{k}\leq CI_{cr}$ **then**    The corresponding combination recorded as an identifiable set**end if**

## 3. Results

In this section, we apply our method to select subsets of practically identifiable parameters in the Chemical Master Equation on three realistic models. We observe that the collinearity indices play a significant role in finding the subsets of estimable parameters, using local stochastic sensitivities. The parametric sensitivities of the stochastic discrete model of well-stirred biochemical systems are approximated by finite-difference schemes, namely the Common Random Number, Common Reaction Path, and Coupled Finite Difference techniques. By applying perturbation in each of these finite-difference techniques, we can assess the sensitivity of the model outputs to changes in the model’s parameters. The choice of perturbation size for finite-difference approximations is essential for obtaining accurate and reliable results while minimizing computational effort. The specific perturbation sizes, representing 5%, 1%, 2% of the parameter value, are often chosen based on a trade-off between accuracy and numerical stability. In addition, we find the parameters with high sensitivities. Those with low sensitivity have a reduced impact on the model outputs and cannot be estimated accurately. In the stochastic context, we consider the SVD of the normalized sensitivity matrix to determine its rank. This rank gives the number of model parameters that are not collinear.

For validation of the methods introduced above, we compare the results obtained with the Chemical Master Equation, with those derived with the Chemical Langevin Equation and those for the reaction rate equations, on two models of biochemically reacting systems. Still, we emphasize the importance of considering stochastic discrete models of biochemical networks to accurately describe the dynamics of these systems, particularly when some molecular populations are small or noise is driving the system behavior. The parametric sensitivities estimated for the reaction rate equations or the Chemical Langevin Equations may not yield accurate estimability results, in general. For each model, we generated 10,000 coupled trajectories to approximate the parametric sensitivities of the Chemical Master Equation by finite-difference schemes. The CFD strategy is considered to be more accurate and reliable than the CRN and the CRP methods [28]. The case studies tested are an infectious disease network [40], the Michaelis–Menten system and a genetic toggle-switch model [11].

### 3.1. Infectious Disease Model

An infectious disease model [40] considers two species: $S_{1}$—the infected particles and $S_{2}$—the particles which can be infected. These species, which may depict molecules, cells, or humans, participate in five reactions. The first two reactions represent the death of species $S_{1}$ and $S_{2}$, respectively, while the third and fourth reactions describe the birth or production of particles of the $S_{1}$ and $S_{2}$ type. The two species interact through the fifth reaction, in which an infected particle $S_{1}$ infects a particle $S_{2}$. The initial conditions are $S_{1}\left(0\right)=20$ and $S_{2}\left(0\right)=40$. The system is studied on the time-interval [0,10]. For our simulations, 10,000 trajectories were generated to estimate the solution of the Chemical Master Equation.

**Figure 1:**
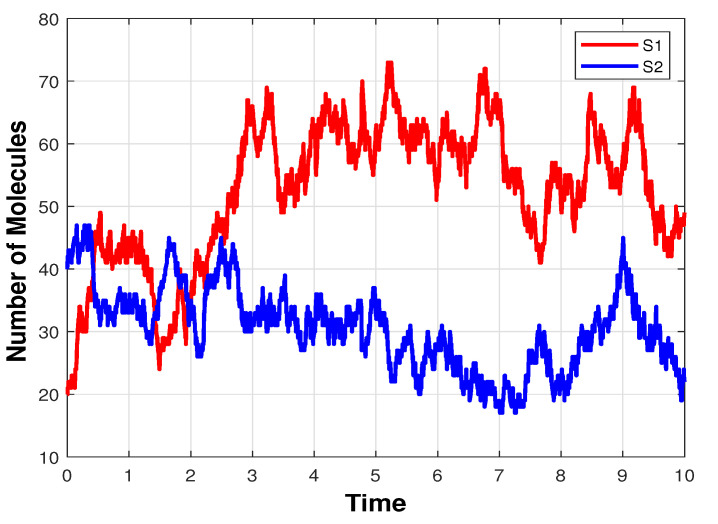
Infectious disease model: the evolution in time of the number of molecules of the species $S_{1}$—infected individuals and $S_{2}$—individuals which can be infected, generated with Gillespie’s algorithm, on the interval [0,10].

Table 1 provides information on the reaction channels of the biochemical system and the values of their rate parameters. It includes the reaction channels denoted by $R_{1}$, $R_{2}$, $R_{3}$, $R_{4}$, and $R_{5}$. Each reaction is described by its reactants and products. The last column lists the parameter values corresponding to the rates at which the reactions occur. These parameter values are specified for the stochastic model considering molecular numbers, rather than for the deterministic reaction rate equations expressed in terms of concentrations. A sample trajectory of the number of the infected $S_{1}$ particles and of the susceptible $S_{2}$ particles as functions of time, computed using Gillepie’s algorithm, is given in Figure 1.

The finite-difference sensitivity estimations are calculated with 10,000 trajectories using the CFD, the CRN, and the CRP strategies, with a perturbation of 5% of the parameter value. The path-wise sensitivities for the Chemical Langevin Equation are computed over 10,000 trajectories, with the Euler-Maruyama scheme applied to the Equations (2) and (7), and are utilized to estimate the sensitivities of the expected value of the state vector. Also, the parametric sensitivities are approximated for the reaction rate equations. These estimations are used to calculate the collinearity indices for all parameter combinations, for the Chemical Master Equation, the Chemical Langevin Equation, and the RRE models. The results are presented in Table 2, Table 3, Table 4, Table 5 and Table 6. The sensitivity measures are reported in Table 2, showing that $c_{2}$ is the least significant among all the parameters.

Table 3, Table 4, Table 5 and Table 6 reveal that the collinearity indices for the reaction rate equation and the Chemical Langevin Equation models exhibit greater consistency with the collinearity indices for the Chemical Master Equation, computed using with the CFD sensitivity estimator, compared to the CRN and the CRP estimators. Notably, the pair subset $\left\{c_{1},c_{3}\right\}$ has the highest collinearity index; however, it is relatively low for the CRP and the CRN schemes in comparison with the other estimations. This is due to the lower accuracy of the CRP and the CRN schemes when compared to the CFD technique. For pair sets, the subset $\left\{c_{1},c_{3}\right\}$, for the triple sets, the subset $\left\{c_{3},c_{4},c_{5}\right\}$ and among the quadruple ones, the subset $\left\{c_{2},c_{3},c_{4},c_{5}\right\}$ have high value of collinearity indices in relation to the other subsets.

There is no subset with high collinearity indices (>20) in pair subsets (Table 3) but there is a parameter subset of size 3 with collinearity index greater than 20 (Table 4). In fact, the parameter subset $\left\{c_{3},c_{4},c_{5}\right\}$ is not identifiable with the Coupled Finite Difference sensitivity estimator, the Chemical Langevin Equation, or the deterministic sensitivities. However, the Common Random Number and the Common Reaction Path sensitivities show different results. In Table 5, two parameter subsets of size 4 show a collinearity index greater than 20 with the deterministic, stochastic continuous, and CFD sensitivity estimations. All subsets containing the parameters $\left\{c_{3},c_{4},c_{5}\right\}$ are collinear, which is in agreement with the results in Table 4. This indicates that these parameter subsets are poorly identifiable. Consequently, the sensitivity-based estimability analysis performed on the RRE, the CLE, and the CME models are in agreement, thus validating the proposed method for the more general discrete stochastic model. The Common Random Number and the Common Reaction Path techniques could not provide an accurate assessment of the identifiability of various subsets, with only 10,000 realizations, being thus less reliable.

### 3.2. Michaelis–Menten Model

**Figure 2:**
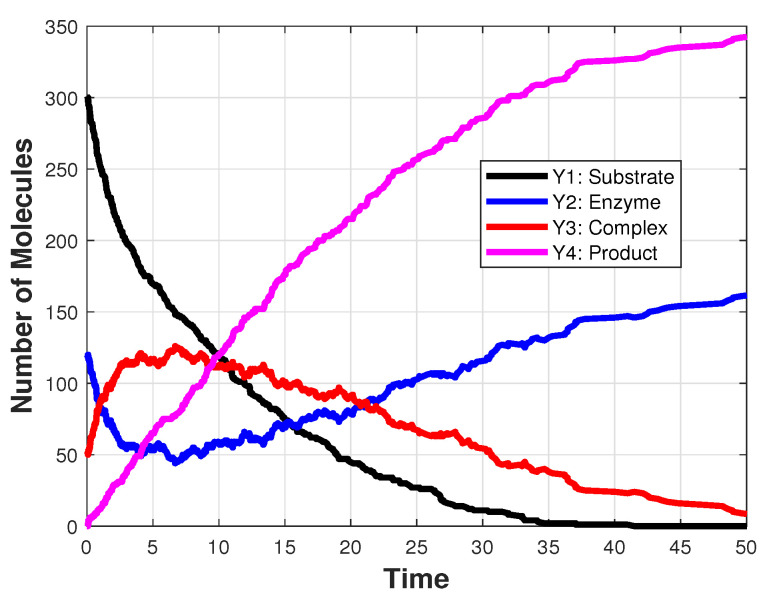
Michaelis–Menten model: the evolution in time of the number of molecules of a substrate, an enzyme, a complex and a product, generated with Gillespie’s algorithm, on the interval [0,50].

The second model we analyze is the Michaelis–Menten biochemical system, which involves four species—a substrate $S_{1}$, an enzyme $S_{2}$, a complex $S_{3}$ and a product $S_{4}$—and three reactions. We denote by $Y_{i}$ the number of molecules of the species $S_{i}$. With this notation, the initial conditions for the number of molecules are $Y_{1}\left(0\right)=\left[5\times 10^{-7}n_{A}vol\right]$, $Y_{2}\left(0\right)=\left[2\times 10^{-7}n_{A}vol\right]$ and $Y_{3}\left(0\right)=Y_{4}\left(0\right)=0$, where $n_{A}=6.023\times 10^{23}$ is Avogadro’s number and $vol=10^{-15}$ denotes the volume of the system. The reactions and the values of the rate parameters are included in Table 7. This model is integrated on the interval [0,50]. Figure 2 depicts a realization of the system state, simulated with Gillespie’s algorithm.

We start by approximating the parametric sensitivities for the Chemical Master Equation. The finite-difference sensitivity estimations obtained with the CFD, the CRP, and the CRN algorithms use a perturbation which represents 1% and 5%, respectively, of the value of the parameter of interest. The sensitivity measures provided in Table 8 indicate that $c_{2}$ may not be estimated as accurately as the other parameters. The collinearity indices obtained for the perturbation value 1% with each sensitivity estimator for pairs of parameters are reported in Table 9, while the indices for the set of all parameters are recorded in Table 10. For each subset, the results for the stochastic Michaelis–Menten model demonstrate low collinearity indices, below 20. The choice of the finite-difference sensitivity estimator does not significantly affect the parameter identifiability. The stochastic discrete modeling approach to identifiability analysis yields parameter subsets that are not collinear for the Michaelis–Menten system. Additionally, the Tables include the RRE identifiability metrics to validate the CME estimability results. The collinearity indices for the perturbation value of 5% can be found in the Appendix A, and they are consistent with the results obtained using a perturbation of 1%.

### 3.3. Genetic Toggle Switch Model

**Figure 3:**
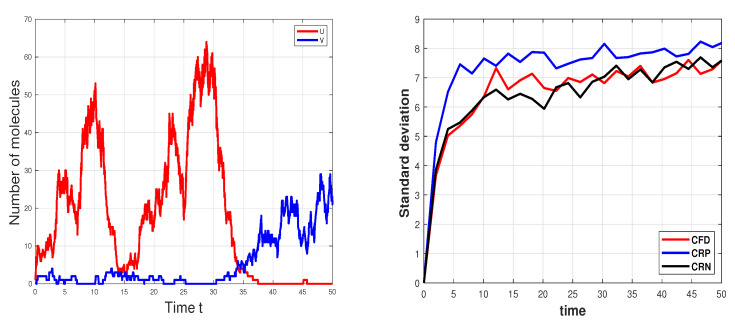
Genetic toggle switch model: (**Left**): the evolution in time of the number of molecules of the species *U* and *V*, generated with Gillespie’s algorithm, on the interval [0,50]. (**Right**): standard deviations of the three estimators, CFD, CRP, and CRN.

The last biochemical system investigated is the genetic toggle switch [11,28]. Multi-stable stochastic switches arise in modeling key biological processes. The model considers two gene pairs, whose interaction creates a bistable switch, as each gene negatively regulates the synthesis of the other gene. Due to the presence of noise, the system can transition between the states represented by an abundance of one species and an almost total absence of the other. In this genetic switch system, the two species *U* and *V* take part in four reactions. Table 11 specifies the reaction channels and their propensities. We examine the system using the following parameter values [11]

(20) $$\alpha_{1}=50,\;\beta =2.5,\;\alpha_{2}=16,\;\gamma =1\;,$$

and the initial conditions $X_{V}\left(0\right)=X_{U}\left(0\right)=0$. Figure 3 displays a sample path for the molecular numbers of the two species, simulated with Gillespie’s algorithm (left) along with the standard deviation of the CFD, CRP, and CRN sensitivity estimators as functions of time (right).

The reaction rate equation model cannot capture the stochastic transitions between the states, and thus the deterministic tools for analyzing this system are not applicable. We perform an estimability analysis of the Chemical Master Equation model for the genetic toggle switch, on the interval [0,50]. To assess how variations in the parameter values affect the dynamics of the system, we approximate the local sensitivities with respect to the parameters whose values are given by (20). We simulate 10,000 coupled sample paths with the CFD, and the CRP methods. The finite-difference sensitivity estimators are applied with a perturbation $\theta =10^{-4}$ for each parameter value. The sensitivity measures are provided in Table 12 and those calculated using the CFD method show that all parameters have $\delta^{msqr}>0.2$, being thus important enough, while the RRE sensitivity measures indicate that the parameters β and γ are insignificant.

Employing the local sensitivity approximations, we compute the collinearity indices for all the subsets of the parameter set $\left\{\alpha_{1},\alpha_{2},\beta ,\gamma \right\}$. Table 13, Table 14 and Table 15 record the collinearity indices for the pair, triple and quadruple subsets, respectively. No subset of parameters exhibits collinearity based on the CFD, the CRP, and the CRN sensitivity estimations. We conclude that all four parameters are identifiable for the stochastic discrete model. These results are confirmed by the singular values computed with the CFD sensitivity estimator, which are [32.21;29;12.18;4]. Different values of the parameters for this model may yield different results for estimability in the stochastic genetic toggle-switch system.

## 4. Discussion

Stochastic models of well-stirred biochemical processes provide a valuable framework for capturing inherent variability at the cellular level when some molecular species have low amounts. Chemical Master Equation is a frequently adopted stochastic discrete model for such processes. By contrast, deterministic approaches are often not suitable for modeling cellular systems as they fail to capture the intrinsic randomness observed experimentally. Many models of realistic biochemical processes depend on a fairly large number of parameters. The values of some of these parameters may be unknown and have to be estimated. Parameter estimation is a critical step in modeling biochemical systems. However, determining appropriate parameter values for stochastic discrete models of biochemical networks poses many challenges. It is essential to determine the key parameters which are identifiable from the experimental data, as well as those that cannot be reliably estimated. For a subset of parameters to be practically identifiable, each parameter of the subset should have a significant contribution to the system dynamics as well as the parameters of the subset should not be correlated.

In this work, we propose a method for detecting collinearity in subsets of parameters for the stochastic discrete model of the Chemical Master Equation, with the goal of finding the parameter sets that exert the greatest influence on the biochemical system state. In addition, we introduce a technique for determining the highest parameter identifiable sets for stochastic biochemical systems, by extending methods from deterministic models to stochastic models. Our analysis is based on estimating the local sensitivities of the system state with respect to the model’s parameters. This is achieved by utilizing finite-difference approximations of the parameter sensitivities, specifically the Coupled Finite Difference, the Common Reaction Path, and the Common Random Number schemes. Furthermore, we examine the role of the singular value decomposition of the sensitivity matrix in identifying parameters that are not collinear in stochastic models of biochemical systems. On one hand, we showed that our practical identifiability method is accurate, by comparing the results obtained in the deterministic and stochastic scenarios, on two biochemical systems of practical importance, for which the deterministic model accurately describes the evolution of the expected value of the stochastic system state. Excellent agreement among the various approaches was obtained for these biochemical networks. On the other hand, we wish to emphasize that, in general, a stochastic strategy for selecting identifiable parameter sets should be considered, as it relies on more accurate and reliable estimations of the parametric sensitivities for the widely applicable model of the Chemical Master Equation, compared to the deterministic reaction rate equations. The advantages of our approach over the deterministic one were illustrated by the tests performed on a third model, a genetic toggle switch system exhibiting an interesting multistable behavior. For this model, our stochastic identifiability strategies display excellent performance, while the deterministic techniques show their limitations, by not being able to assess the estimability of the model parameters.

We expect the method to perform best on stochastic biochemical models with a moderate number of reaction rate parameters. Specifying identifiable parameter subsets with the tools provided above may be used to refine models, improve predictions, and study the underlying biological processes under consideration.

## Figures and Tables

**Table 1:** Infectious disease model: the list of reactions and the corresponding rate parameter values.

	Reaction Channel	Rate Parameter Value
$R_{1}$:	$S_{1}⟶\emptyset$	$c_{1}=2.0$
$R_{2}$:	$S_{2}⟶\emptyset$	$c_{2}=0.1$
$R_{3}$:	$\emptyset ⟶S_{1}$	$c_{3}=25$
$R_{4}$:	$\emptyset ⟶S_{2}$	$c_{4}=75$
$R_{5}$:	$S_{1}+S_{2}⟶S_{1}+S_{1}$	$c_{5}=0.05$

**Table 2:** Infectious disease model: comparison of $\delta^{msqr}$ for a 5% perturbation.

Parameter	$\delta^{\mathrm{msqr}}$ of CFD Sensitivity	$\delta^{\mathrm{msqr}}$ of CRP Sensitivity	$\delta^{\mathrm{msqr}}$ of CRN Sensitivity	$\delta^{\mathrm{msqr}}$ of RRE Sensitivity	$\delta^{\mathrm{msqr}}$ Path-Wise Sensitivity
$c_{1}$	0.97	0.96	0.94	0.97	0.98
$c_{2}$	0.02	0.02	0.1	0.02	0.02
$c_{3}$	0.26	0.29	0.26	0.26	0.26
$c_{4}$	0.55	0.66	0.54	0.55	0.55
$c_{5}$	0.68	0.69	0.67	0.71	0.71

**Table 3:** Infectious disease model: collinearity indices for pair subsets. The CME sensitivities are estimated over 10,000 trajectories with the CFD, CRN, and CRP algorithms and a 5% perturbation.

Parameter Subset	Collinearity Index of CFD Sensitivity	Collinearity Index of CRP Sensitivity	Collinearity Index of CRN Sensitivity	Collinearity Index of RRE Sensitivity	Collinearity Index of Path-Wise Sensitivity
$c_{4}$ $c_{5}$	1.18	1.13	1.18	1.2	1.19
$c_{3}$ $c_{5}$	1.93	1.17	1.94	1.92	1.95
$c_{3}$ $c_{4}$	1.339	1.25	1.32	1.32	1.31
$c_{2}$ $c_{5}$	1.103	1.15	1.13	1.18	1.17
$c_{2}$ $c_{4}$	4.69	2.37	1.16	9.77	9.96
$c_{2}$ $c_{3}$	1.43	1.27	1.02	1.34	1.33
$c_{1}$ $c_{5}$	1.86	1.89	1.9	1.85	1.86
$c_{1}$ $c_{4}$	1.35	1.28	1.33	1.34	1.34
$c_{1}$ $c_{3}$	10.816	3.04	7.2	11.34	11.22
$c_{1}$ $c_{2}$	1.466	1.31	1.00	1.36	1.35

**Table 4:** Infectious disease model: collinearity indices for triple subsets. The CME sensitivities are estimated over 10,000 trajectories with the CFD, CRN, and CRP algorithms and a 5% perturbation.

Parameter Subset	Collinearity Index of CFD Sensitivity	Collinearity Index of CRP Sensitivity	Collinearity Index of CRN Sensitivity	Collinearity Index of RRE Sensitivity	Collinearity Index of Path-Wise Sensitivity
$c_{3}$ $c_{4}$ $c_{5}$	21.19	2.63	9.6	21.3	21.77
$c_{2}$ $c_{4}$ $c_{5}$	5.0444	2.38	1.2	9.97	10.15
$c_{2}$ $c_{3}$ $c_{5}$	7.7768	2.91	2.01	10.48	10.51
$c_{2}$ $c_{3}$ $c_{4}$	4.88	2.38	1.43	9.83	10.01
$c_{1}$ $c_{4}$ $c_{5}$	9.92	3.65	9.4	10.83	10.98
$c_{1}$ $c_{3}$ $c_{5}$	11.07	3.12	7.2	11.68	11.73
$c_{1}$ $c_{3}$ $c_{4}$	10.87	3.05	7.2	11.46	11.45
$c_{1}$ $c_{2}$ $c_{5}$	7.44	4.8	2	7.87	7.95
$c_{1}$ $c_{2}$ $c_{4}$	4.95	2.38	1.43	9.82	10.01
$c_{1}$ $c_{2}$ $c_{3}$	11.02	3.06	7.3	11.45	11.44

**Table 5:** Infectious disease model: collinearity indices for quadruple subsets. The CME sensitivities are estimated over 10,000 trajectories with the CFD, CRN, and CRP algorithms and a 5% perturbation.

Parameter Subset	Collinearity Index of CFD Sensitivity	Collinearity Index of CRP Sensitivity	Collinearity Index of CRN Sensitivity	Collinearity Index of RRE Sensitivity	Collinearity Index of Path-Wise Sensitivity
$c_{1}$ $c_{2}$ $c_{3}$ $c_{4}$	11.509	3.06	7.3	11.53	11.49
$c_{1}$ $c_{2}$ $c_{3}$ $c_{5}$	11.092	4.88	7.3	13.65	13.53
$c_{1}$ $c_{2}$ $c_{4}$ $c_{5}$	10.2347	4.94	9.4	13.82	14.20
$c_{1}$ $c_{3}$ $c_{4}$ $c_{5}$	22.6313	3.87	10.54	22.19	22.49
$c_{2}$ $c_{3}$ $c_{4}$ $c_{5}$	21.4369	2.91	9.6	25.71	27.77

**Table 6:** Infectious disease model: collinearity indices for the set of all kinetic parameters. The CME sensitivities are estimated over 10,000 trajectories with the CFD, CRN, and CRP algorithms and a 5% perturbation. The singular values for the CFD, the CLE, and the RRE sensitivity estimations show that the number of parameters that are not collinear is four.

Parameter Subset	Collinearity Index of CFD Sensitivity	Collinearity Index of CRP Sensitivity	Collinearity Index of CRN Sensitivity	Collinearity Index of RRE Sensitivity	Collinearity Index of Path-Wise Sensitivity
$c_{1}$ $c_{2}$ $c_{3}$ $c_{4}$ $c_{5}$	22.65	5.01	10.54	26.17	28.09
singular values	16.31, 9.48,	16.27, 10.35,	15.86, 9.28,	36.73, 21.86,	37.03, 21.76,
	1.06, 0.21, 0.06	2.98, 1.79, 0.14	1.31, 1.1, 0.52	2.21, 0.48, 0.09	2.19, 0.48, 0.09

**Table 7:** Michaelis–Menten model: the list of reactions and the corresponding rate parameter values.

	Reaction Channel	Rate Parameter Value
$R_{1}$:	$S_{1}+S_{2}⟶S_{3}$	$c_{1}=10^{6}/\left(n_{A}vol\right)$
$R_{2}$:	$S_{3}⟶S_{1}+S_{2}$	$c_{2}=10^{-4}$
$R_{3}$:	$S_{3}⟶S_{4}+S_{2}$	$c_{3}=10^{-1}$

**Table 8:** Michaelis–Menten model: comparison of $\delta^{msqr}$ for a 1% perturbation.

Parameter	$\delta^{\mathrm{msqr}}$ of CFD Sensitivity	$\delta^{\mathrm{msqr}}$ of CRP Sensitivity	$\delta^{\mathrm{msqr}}$ of CRN Sensitivity	$\delta^{\mathrm{msqr}}$ of RRE Sensitivity
$c_{1}$	1.11	1.1	1.07	1.07
$c_{2}$	0.002	0.01	0.003	0.002
$c_{3}$	1.31	1.30	1.29	1.29

**Table 9:** Michaelis–Menten model: collinearity indices for pair subsets. The CME sensitivities are estimated over 10,000 trajectories with the CFD, CRN, and CRP algorithms and a 1% perturbation.

Parameter Subset	Collinearity Index of CFD Sensitivity	Collinearity Index of CRP Sensitivity	Collinearity Index of CRN Sensitivity	Collinearity Index of RRE Sensitivity
$c_{1}$ $c_{2}$	2.9	1.35	1.47	4.85
$c_{1}$ $c_{3}$	2.21	2.17	2.17	2.17
$c_{2}$ $c_{3}$	1.56	1.21	1.2	1.87

**Table 10:** Michaelis–Menten model: collinearity indices for the triple subset. The CME sensitivities are estimated over 10,000 trajectories with the CFD, CRN, and CRP algorithms and a 1% perturbation.

Parameter Subset	Collinearity Index of CFD Sensitivity	Collinearity Index of CRP Sensitivity	Collinearity Index of CRN Sensitivity	Collinearity Index of RRE Sensitivity
$c_{1}$ $c_{2}$ $c_{3}$	3.92	2.25	2.43	5.3

**Table 11:** Genetic toggle switch model: the list of reactions and their propensity functions.

	Reaction Channel	Propensity Function
$R_{1}$:	∅⟶U	$a_{1}=\frac{\alpha_{1}}{1+X_{V}^{\beta }}$
$R_{2}$:	U⟶∅	$a_{2}=X_{U}$
$R_{3}$:	∅⟶V	$a_{3}=\frac{\alpha_{2}}{1+X_{U}^{\gamma }}$
$R_{4}$:	V⟶∅	$a_{4}=X_{V}$

**Table 12:** Genetic toggle switch model: comparison of $\delta^{msqr}$.

Parameter	$\delta^{\mathrm{msqr}}$ of CFD Sensitivity	$\delta^{\mathrm{msqr}}$ of RRE Sensitivity
α1	2.22	0.89
β	0.6762	0
α2	4.21	0.31
γ	4.3	0

**Table 13:** Genetic toggle switch model: collinearity indices for pair subsets.

Parameter Subset	Collinearity Index of CFD Sensitivity	Collinearity Index of CRP Sensitivity	Collinearity Index of CRN Sensitivity	Collinearity Index of RRE Sensitivity
$\alpha_{1}$ $\alpha_{2}$	1	1.01	1.72	2.22
γ $\alpha_{2}$	1.32	1.08	1.12	*
γ $\alpha_{1}$	1.27	1.17	1.07	*
β $\alpha_{2}$	1.01	1.1	1.56	*
β $\alpha_{1}$	1.00	1.35	2.13	*
β γ	1.19	1.25	1.1	*

The CME sensitivities with respect to parameters are estimated over 10,000 with the CFD and CRP methods and perturbation $\theta =10^{-4}$. *: Collinearity index does not exist.

**Table 14:** Genetic toggle switch model: collinearity indices for triple subsets.

Parameter Subset	Collinearity Index of CFD Sensitivity	Collinearity Index of CRP Sensitivity	Collinearity Index of CRN Sensitivity	Collinearity Index of RRE Sensitivity
β γ $\alpha_{1}$	1.38	1.37	2.46	*
β γ $\alpha_{2}$	1.42	1.25	1.80	*
β $\alpha_{1}$ $\alpha_{2}$	1.01	1.38	2.18	*
γ $\alpha_{1}$ $\alpha_{2}$	1.52	1.19	1.73	*

The CME sensitivities with respect to parameters are estimated over 10,000 with the CFD and CRP methods and perturbation $\theta =10^{-4}$. *: Collinearity index does not exist.

**Table 15:** Genetic toggle switch model: collinearity indices for the quadruple subset.

Parameter Subset	Collinearity Index of CFD Sensitivity	Collinearity Index of CRP Sensitivity	Collinearity Index of CRN Sensitivity	Collinearity Index of RRE Sensitivity
β γ $\alpha_{1}$ $\alpha_{2}$	1.64	1.39	2.45	*

The CME sensitivities with respect to parameters are estimated over 10,000 with the CFD and CRP methods and perturbation $\theta =10^{-4}$. *: Collinearity index does not exist.

## Data Availability

No new data were created.

## Appendix A

**Table A1:** Michaelis–Menten model: comparison of $\delta^{msqr}$ for a 5% perturbation.

Parameter	$\delta^{\mathrm{msqr}}$ of CFD Sensitivity	$\delta^{\mathrm{msqr}}$ of CRP Sensitivity	$\delta^{\mathrm{msqr}}$ of CRN Sensitivity	$\delta^{\mathrm{msqr}}$ of RRE Sensitivity
$c_{1}$	1.03	1.04	1.04	1.07
$c_{2}$	0.002	0.005	0.003	0.002
$c_{3}$	1.22	1.23	1.23	1.29

**Table A2:** Michaelis-Menten model: collinearity indices for pair subsets. The CME sensitivities are estimated over 10,000 trajectories with the CFD, CRN and CRP algorithms and a 5% perturbation.

Parameter Subset	Collinearity Index of CFD Sensitivity	Collinearity Index of CRP Sensitivity	Collinearity Index of CRN Sensitivity	Collinearity Index of RRE Sensitivity
$c_{1}$ $c_{2}$	3.43	2.18	2.59	4.85
$c_{1}$ $c_{3}$	2.21	2.13	2.13	2.17
$c_{2}$ $c_{3}$	1.67	1.48	1.49	1.87

**Table A3:** Michaelis–Menten model: collinearity indices for the triple subset. The CME sensitivities are estimated over 10,000 trajectories with the CFD, CRN and CRP algorithms and a 5% perturbation.

Parameter Subset	Collinearity Index of CFD Sensitivity	Collinearity Index of CRP Sensitivity	Collinearity Index of CRN Sensitivity	Collinearity Index of RRE Sensitivity
$c_{1}$ $c_{2}$ $c_{3}$	4.08	2.78	3.4	5.3
